# Book Review

**Published:** 1927

**Authors:** 


### Reviews of Books.

**Diseases of the Nose, Throat and Ear.** Second edition. By several authors. Editor: A. Logan Turner, M.D., LL.D., F.R.C.S.E. Pp. xxiv., 440. 234 illustrations and 12 plates. Bristol: J. Wright & Sons Ltd. 1927. Price 20s.—It is seldom that we find a volume that merits unstinted praise, which sends us hunting the dictionary for superlatives. Such is this. Under an inch and a half thick, in octavo, it gives a comprehensive survey of its subject. The commoner affections are well described, with their course and treatment; considerable detail of operative procedure is available to the reader. The most modern methods are discussed, and even the most difficult and recondite questions find their place: for example, the differential diagnosis between a tumour of the cerebellum and one of the eighth nerve. The illustrations are numerous, well chosen and excellently produced, the typography makes for pleasant reading and a complete index facilitates reference. The second edition is in every way a worthy successor to the first.

**The Ear, Nose and Throat in General Practice.** By D. A. Crow, M.B. Pp. x., 150. 44 figures. Oxford University Press. 1927. Price 10s. 6d.—It is curious that in the title of a book the phrase "in general practice" should still be thought a sufficient indication of superficial treatment. This volume really consists of six short essays on as many aspects of its subject, and a chapter, aptly named "Miscellaneous," which mentions some previous omissions. Its main fault is lack of balance. More than a quarter of the book is devoted to three methods of removing tonsils. "The Nose" receives less space than the fifteen pages allotted to endoscopic methods for foreign bodies in the gullet, etc. Chronic otorrhœa receives no mention at all, but paralysis of the œsophagus, one of the rarest conditions known, is described. The author is frankly pessimistic on the subject of deafness. With his insistence on the value of early drainage in acute otitis we agree, but to maintain that paracentesis is the only treatment appears a *reductio ad absurdum*. As an exposition of certain personal views and feelings the book is not without interest to the specialist; as a guide to the general practitioner it is wholly inadequate.

**Rheumatic Diseases.** By MATTHEW B. RAY, D.S.O., M.D. Pp. 91. London: Kegan Paul. 1927. Price 2s. 6d.—The author has condensed into the small space of one of the "Psyche Miniatures" a good deal of information about the nature and treatment of the "rheumatic" diseases. He has wisely chosen to arrange his matter under the classification adopted by the Ministry of Health in its report on those diseases, except that he has utilised the terms "rheumatoid arthritis, atrophic" and "infective peri-arthritis" to describe what he considers allied but distinct forms of peri-articular disease. The book is a sane and balanced summary of a subject which is much to the fore at the present time.

**Orthopædic Surgery.** By W. A. COCHRANE, F.R.C.S.E. Pp. xxiii., 528. Illustrated. Edinburgh: E. & S. Livingstone. 1926. Price 21s.—The author of this volume represents the best teaching of the Scottish schools combined with the famous Boston orthopædic school. The ideals aimed at in this book are very high, namely the teaching of broad principles with the addition of special knowledge. Anatomy and physiology are given great prominence as the foundation, and the stress laid upon basic conceptions is admirable. The recognition of predisposition to deformity, and its prevention, are the first tasks of every medical man in general practice as well as of the orthopædic specialist. These important subjects are fully discussed in connection with the anatomy and physiology of crippling conditions. The first part of the book deals with posture, tone, physical education and reconstruction. The second is devoted to the different parts of the locomotor apparatus. The regional arrangement is certainly convenient for reference, but does not lend itself to a scientific exposition of broad principles. The author does, fortunately, deal with poliomyelitis and with spastic paralysis in a broad manner, but should have placed these matters early in the book, and not after the regional sections, in which there is frequent need for reference to them. The subject of fractures also suffers severely from the fact that the regional classification is always round the joints. So that, for example, a fracture of the shaft of the femur receives scanty mention, while fractures of neck and condyles are only briefly described. All the ordinary deformities of the joints and of the spine are well described and profusely illustrated; this part of the book will be of the greatest value to student and practitioner.

**Physical Signs in Clinical Surgery.** By Hamilton Bailey, F.R.C.S. Pp. xv., 217. 261 illustrations. Bristol: J. Wright & Sons Ltd. 1927. Price 21s.—Nothing but praise can be given to this excellent little manual. It should be in the possession of every student starting his dressership. It will also be found extremely useful to tutors whose duty it is to teach elementary surgery. The use of the book is explained by its title. It is so well illustrated that the text is almost superfluous. At the same time the latter is clearly and concisely written, making it a pleasure to read. The photographs, which are the chief form of illustration, are excellently reproduced. The coloured photograph of traumatic asphyxia is indeed a work of art. Mistakes are difficult to find, but in Fig. 228 the knee joint should be shown fully extended instead of partly flexed. In the facsimile records the author's handwriting is a little difficult to read. The publishers are to be congratulated on their high standard of production.

**Perspective in Relation to X-ray Interpretation.** By William Cotton, M.A., M.D. Third edition. Pp. 8. Illustrated. Manchester: Jesse Broad & Co. Ltd. Price 10s. 6d.—This little pamphlet enshrines the views that the author has already expressed upon numerous occasions as to the proper interpretation of skiagrams. There is undoubtedly much truth in what he says, though we do not feel that the matter is so obscure or so little recognised as does Dr. Cotton. The practitioner who constantly uses skiagraphy learns to think in terms of shadow, and to appreciate the apparent distortion of parts at its true value; few indeed would turn to Euclid, Books v. and vi. or Helmholtz's *Optics in Relation to Painting*, as the author recommends.

**A Manual of General Medical Practice.** By W. Stanley Sykes, M.A., M.B., D.P.H. Pp. xi., 216. London: H. K. Lewis & Co. 1927. Price 7s. 6d.—This is a book for beginners, whom it will help. The author presents the features of disease in clear outline; for example, influenza, which he studied in the epidemic of 1924. The initial stages of appendicitis can be puzzling, and the censure of delay in diagnosis undeserved. Dr. Sykes finds the pulse a better guide than the temperature. He maintains that of all hospital appointments the most valuable to the general practitioner is the obstetric; and argues that however great the convenience of the hospital, the majority of the population are born, live, lie sick and die in their own homes.

**An Introduction to the Law and Tradition of Medical Practice.** By WM. SANDERSON, M.A., LL.B., and E. B. A. RAYNER, B.A., LL.B. Pp. xii., 77. London: H. K. Lewis & Co. 1926. Price 7s. 6d.—The authors have attempted in this small volume of 72 pages to cover the salient facts regarding (1) the ethics of the profession, and (2) the law as it relates to the liabilities and privileges of medical practice. A perusal of this handbook, however, shows that they are on much surer ground as lawyers in the section on law than as laymen on the subject of ethics, and the number of errors suggest that they would have been better advised to secure the collaboration of a medical man in dealing with this subject. To take one instance only, on page 23 occurs the following: "It is customary for one who practises surgery only to style himself Mr. A. B., Surgeon." They may haunt the purlieus of Cavendish Square in vain for the plate of a consulting surgeon that bears anything more than his name. Apart from these inaccuracies the book is worth purchasing for its legal summary, and might well find a place in the library of any medical man.

**The Diagnosis of Pancreatic Disease.** By ROBERT COOPE, M.D., B.Sc., M.R.C.P. Pp. viii., 112. London: Oxford University Press. 1927. Price 5s. net.—There is no organ of the body the diseases of which present such difficulties in diagnosis as those of the pancreas. In this short work Dr. Coope, after dealing with the anatomy and physiology of the gland, reviews the various methods of examination in pancreatic disease, clinical observation, X-ray findings, and attempts to detect failure of discharge of the juice into the duodenum. He also discusses failure of insulin production and criticises adversely the numerous indirect tests of failure of pancreatic secretion. He attaches the greatest importance to the detection in the stools of undigested muscle fibres, and gives clear and precise directions for the carrying out of this test. The author dismisses as unreliable such reactions as Loewi's and Cammidge's tests. Undoubtedly the book will be found most useful to the clinician in simplifying and clarifying the work of investigating diseases of the pancreas.

**Malaria in India.** By Major-General Sir PATRICK HEHIR, K.C.I.E., C.B., C.M.G., M.D. Pp. xix., 490. Illustrated. Oxford University Press. 1927. Price £2 2s. net.—This book has had a chequered career. In his preface the author tells us that during the War he lost two large boxes of MS., with many hundreds of illustrations of which it has been impossible to secure copies. Although he does not say so, we imagine that he owes this disappointment to his share in the heroic defence of Kut-el-Amara and his subsequent experiences as a prisoner of war with the Turks. At all events the book that has finally emerged is one of which its author has reason to be proud. He has faced successfully the formidable task of surveying one of the most massive problems to which man has ever devoted his energies—the conquest of malaria. The magnitude of the problem is to be gauged from an estimate, apparently well founded, of the incidence of malaria in India, on p. 12, to the effect that the number of cases of malaria in India calling for treatment is 100,000,000 per annum. To this must be added another calculation, namely, that it is improbable that more than a fifth of affected persons ever take quinine. Almost the whole book is devoted to a consideration of the ætiology of the disease and its prevention, only a short clinical section being considered necessary. Sir Patrick Hehir quotes with approval Manson's dictum that "in serious cases (of malaria), to use any other drug to the exclusion of quinine is culpable trifling," adding that the word "quinine" must be read as a synonym for the crystallisable alkaloids of cinchona. But the aspect of the book which is of most interest is that which reveals this problem of reducing the malaria of India as one which affects every phase of Indian life. The author says of this that it is a task beyond the powers of any government. If it is to be successfully undertaken it must be by the people themselves. The plan of campaign necessary to achieve this purpose is outlined by the author in pages that no one but an expert would venture to criticise. It is obvious to any reader, however, that his propositions are based on sincerity of purpose as well as on wide experience, and we believe that the book will prove valuable not only to the medical profession but also to everyone interested in the welfare of India.

